# The Effect of Substrate Surface Oxidation on Patterned Graphene Growth for Flexible Electronics

**DOI:** 10.3390/ma18143338

**Published:** 2025-07-16

**Authors:** Ruiqi Zhang, Ning Hou, Huawen Wang, Xu Chen, Haofei Shi, Xin Li

**Affiliations:** 1 Chongqing Institute of Green and Intelligent Technology, Chinese Academy of Sciences, Chongqing 400714, China; zhangruiqi@cigit.ac.cn (R.Z.);; 2 Chongqing School, University of Chinese Academy of Sciences, Chongqing 400714, China; 3 Materials Science and Engineering School, Chongqing Jiaotong University, Chongqing 400074, China

**Keywords:** patterned graphene, high precision, flexible electronics, in situ fabrication, substrate surface oxidation, boundary precision, feature size, quenching effect

## Abstract

Graphene exhibits exceptional electronic properties, superior mechanical strength, and remarkable flexibility, driving significant advances in flexible electronics. However, achieving high-precision patterned graphene via in situ fabrication for such applications remains challenging, limiting the development of graphene-based flexible devices. In this study, we successfully synthesized patterned graphene with high precision by substrate surface oxidation technology. The effect of substrate surface oxidation on patterned graphene growth was deeply investigated. By regulating the oxidation time, we precisely controlled the oxidation degree of the substrate and characterized the boundary precision between oxidized and unoxidized regions. Finally, we achieved the high-precision in situ fabrication of patterned graphene with a feature size of 0.5 μm on selectively oxidized substrates. Furthermore, we fabricated a flexible fluorescent device based on patterned graphene, demonstrating the pronounced fluorescence quenching effect of graphene (*I*_Gr-free_/*I*_Gr-cov_ ≈ 3).

## 1. Introduction

In recent years, flexible electronic devices have attracted increased attention [1,2,3,4,5]. Graphene, a two-dimensional material [6], possesses impressive carrier mobility of 2 × 10^5^ cm^2^·V^−1^·S^−1^, exceptional breaking strength of 42 N·m^−1^, thermal conductivity exceeding 5300 W·m^−1^·K^−1^, excellent optical transmittance of 97.7% per layer, and good flexibility [7,8,9,10,11]. These attributes have propelled graphene to the forefront of applications in flexible electronic devices [5,12,13,14,15,16]. A critical prerequisite for the integration of graphene into flexible electronics lies in achieving precise micro-patterning [17]. Traditionally, photolithography combined with selective etching has been the prevalent method for patterning, involving complex chemical processing [18,19]. However, the complexity of photolithographic workflows introduces residual contaminants and mechanical damage to the substrate, thereby degrading the graphene quality. Another novel and versatile approach, electrochemical nanolithography (EnL), enables the patterning of two-dimensional materials [20]. This technique integrates scanning probe lithography (SPL) [21] with lithographically controlled wetting technology (LCW) [22], where the controlled scanning of conductive probes facilitates localized electrochemical reactions for material modification, thereby achieving precise patterning fabrication. While the EnL method enables the nanoscale-precision patterning of two-dimensional materials, its relatively low throughput presents challenges for large-scale fabrication processes. The direct growth of patterned graphene eliminates the defects and impurities inherent in traditional photolithographic methods, enabling the simultaneous preparation of high-quality graphene and large-scale production.

In our previous work, we demonstrated the feasibility of the selective area reconstruction (SAR) method for the in situ growth of patterned graphene on copper [23]. By utilizing selective oxidation and high-temperature reduction technologies, this method could effectively regulate the surface characteristics of the copper substrate and precisely control the nucleation and growth behavior of the customized graphene structure. Arbitrary precise patterns (feature size of 1 μm) with a high monolayer rate and low defect density can be fabricated by SAR technology. The development of flexible electronics has put forward higher requirements for the precision of patterned graphene.

Therefore, we investigated the effect of substrate surface oxidation on patterned graphene growth. By employing a low-concentration oxidant, we conducted a detailed investigation into substrate surface oxidation. Through optimized selective oxidation on a substrate, we successfully achieved patterned graphene with high precision, with a feature size of 0.5 μm. Raman spectroscopy and 2D peak mapping of the graphene exhibited a low defect density, indicating good crystallinity. Moreover, the flexible photoluminescent fabricated by transferring the patterned graphene onto a polyethylene terephthalate (PET) substrate demonstrated the fluorescence quenching effect of graphene, significantly reducing the original fluorescence intensity to one-third of its initial value.

## 2. Materials and Methods

### 2.1. Pretreatment of Copper Foil

Industrial copper foil (46 μm thickness, CHINALCO Shanghai Copper Company, Shanghai, China) was preprocessed to remove impurities and surface residues. Initially, the copper sheet was cut into 6 × 6 cm^2^ square samples. The square samples were sequentially immersed in acetone and ethanol for 10 min to eliminate residues from the cold-rolling process. The sample was rinsed with ultrapure deionized water to remove alcohol and blow-dried with nitrogen gas. Electrochemical polishing was then performed in phosphoric acid (H_3_PO_4_) under a constant potential (1 V) for 20 min. The polished substrate was rinsed with ultrapure deionized water to remove residual H_3_PO_4_ and dried with nitrogen gas to ensure a contaminant-free surface.

### 2.2. Patterning of Copper Substrate

The process began with spin-coating a photoresist layer (S1800 series, The Dow Chemical Company, Midland, MI, USA) onto the polished copper foil. The spin-coating parameters were set at 300 rpm for 3 s, followed by 1800 rpm for 35 s and finally 3000 rpm for 5 s. The coated foil was baked at 100 °C for 10 min on a hot plate. The copper foil was then exposed through a mask aligner (ABM/6/350/NUV/DCCD/BSV/M, ABM-USA Incorporated company, San Jose, CA, USA) for 15 s using the customized Chongqing Institute logo mask, followed by immediate immersion in AZ 300 MIF developer (Clariant Corporation, Basel, Switzerland) for 60 s to remove the exposed photoresist. Selective oxidation was achieved by applying 5% H_2_O_2_ (in deionized water, Chongqing Chuandong Chemical, Chongqing, China) on the copper foil to form a Cu_x_O/Cu patterned structure. Finally, the sample was soaked in acetone to completely the remove the residual photoresist.

### 2.3. Growth of Patterned Graphene

Patterned graphene was synthesized using an atmospheric pressure chemical vapor deposition (APCVD) system (BTF-1200C, Anhui BEQ Equipment Technology, Hefei, China) with methane as the carbon precursor. The sample was first placed in a 100-cm-diameter heating furnace and heated from room temperature to 1050 °C under a 500 sccm Ar (99.999% purity, Chongqing Lituo Gas Co., Ltd., Chongqing, China) gas flow. The heating rate was set to ramp from room temperature to 800 °C in 30 min (26 °C/min) and then from 800 °C to 1050 °C in another 30 min (8 °C/min). Upon reaching 1050 °C, 50 sccm H_2_ (99.999% purity, Chongqing Lituo Gas Co., Ltd.) was introduced to initiate the annealing process, which lasted for 90 min. Following this, graphene growth was initiated by introducing a 10 sccm CH_4_/H_2_ gas mixture (99% hydrogen and 1% methane, Chongqing Lituo Gas Co., Ltd.), with the growth duration ranging from 0 to 20 min. Heating was immediately terminated upon completion of growth, and forced cooling was implemented using fans. The temperature decreased from 1050 °C to room temperature in approximately 30 min (35 °C/min), while maintaining a 500 sccm Ar flow as a protective atmosphere. All processes were conducted under ambient conditions (25 °C, 50% humidity).

### 2.4. Transfer of Graphene and Device Fabrication

A 6 wt% PMMA/ethyl lactate solution (99.8% purity) was spin-coated as a protective layer. The spin-coating parameters were set at 500 rpm for 5 s, followed by 3000 rpm for 30 s. The sample was then baked in air at 120 °C for 10 min. The PMMA-coated sample was immersed in 1 M FeCl_3_ aqueous solution for 120 min to etch the copper substrate. Following copper etching, the PMMA–graphene film remained floating on the FeCl_3_ solution. The freestanding film was transferred into deionized water using a polyethylene terephthalate (PET) plate for 30 min of rinsing to remove residual metal ions. After rinsing with deionized water, the floating PMMA–graphene film was transferred onto a polyethylene terephthalate (PET) substrate and air-dried at room temperature for 6 h. The thoroughly dried PMMA–graphene–PET sample was immersed in acetone for 30 min to completely dissolve the PMMA layer. Following PMMA removal with acetone, a Super Yellow (SY) fluorescent material was spin-coated to fabricate the photoluminescent device. The chemical reagent Super Yellow powder was purchased from Sigma-Aldrich (St. Louis, MO, USA) (commercial name: Super Yellow light-emitting PPV copolymer, CAS Number: 26009-24-5). The as-received SY powder was dissolved in toluene (2 mg/mL) for spin-coating application as a luminescent material. SY was dissolved in toluene using magnetic stirring at an 800 rpm rotation speed and a temperature of 50 °C. The complete dissolution of SY typically requires at least 12 h.

### 2.5. Measurements and Characterization

Optical microscopy was performed using an MX6R system (Olympus, Tokyo, Japan). Raman spectra were acquired with a 532 nm laser (inVia Reflex, Renishaw, Pune, India). The surface morphology and composition were analyzed by SEM/EDS (JEOL 7640, Tokyo, Japan) and AFM (Bruker Dimension Edge, Berlin, Germany). UV illumination was provided by a ZF-20D system (Shanghai Jinqi Equipment, Shanghai, China), with spectral analysis conducted on an F-7000 spectrophotometer (Hitachi, Tokyo, Japan). The graphene coverage was statistically calculated using the ImageJ software (Version 1.53t). A set of micrographs showing patterned graphene at different growth times was imported into the ImageJ software. The white areas represented the regions where graphene had been grown, while the red areas indicated the oxidized copper substrate. In the software, the images were converted into grayscale images and thresholds were applied to quantify the graphene coverage. Graphene coverage statistics were obtained for samples with different growth times (0–20 min, with an interval of 1 min). Three replicates were measured per time point (n = 3), with the mean coverage and standard deviation calculated for each one. For the data, the statistical method of nonlinear regression was used, the Boltzmann equation was iterated using the Levenberg–Marquardt algorithm, and the number of iterations was 7. The reduced chi-squared value after nonlinear fitting was 5.05494, the residual sum of squares was 80.87903, the coefficient of determination (COD) was 0.99736, and the adj. R-square was 0.99686, achieving fitting convergence. The statistical software used was Origin (OriginLab, Version 10.1.5.132). Raman mapping was performed using a He/Cd laser excitation source (532 nm wavelength, inVia Reflex, London, UK) operating at 1 mW power. A 50× objective lens was employed for measurements. Graphene 2D peak mapping was conducted by setting the scanning peak position at 2700 cm^−1^. A 30 × 30 μm square scanning area was selected with a spatial resolution of 0.5 μm, resulting in a total of 1800 sampling points. This comprehensive mapping required approximately 10 h for completion. Acquired spectral data were converted into a gridded XYZ matrix using the Origin software, and mapping images were subsequently generated through MATLAB (Version R2024b).

## 3. Results and Discussion

### 3.1. Process of Device Fabrication and Characterization of Patterned Substrate

**Figure 1:**
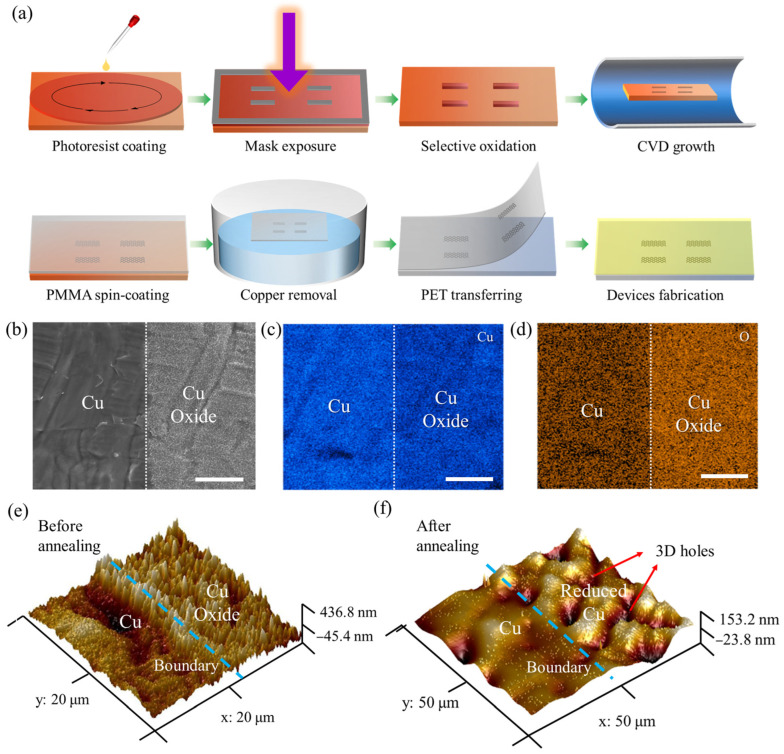
(**a**) Schematic diagram of the fabrication procedure for flexible patterned graphene devices; (**b**) SEM image of copper substrate after selective oxidation for 30 min, scale bar: 5 μm; (**c**,**d**) EDS mappings of copper and oxygen elements taken from (**b**), respectively; (**e**) 3D AFM image of selectively oxidized substrate before annealing; (**f**) 3D AFM image of selectively oxidized substrate after annealing, with red arrows indicating 3D holes.

The fabrication process of the flexible patterned graphene devices is shown in Figure 1a. The procedure involved several steps: photoresist spin-coating, mask exposure, selective oxidation, graphene growth, PMMA spin-coating, substrate dissolution, PET transfer, and flexible luminescent device fabrication. The detailed protocols for each step are provided in the Materials and Methods section. Figure 1b presents an SEM image of the copper substrate after 30 min of selective oxidation. The boundary between copper and copper oxide was clearly visible. The right side of the image appeared gray, revealing the significant enrichment of oxygen elements and the formation of copper oxide. The corresponding elements, copper and oxygen, were analyzed by EDS mapping, as shown in Figure 1c,d, respectively. The concentration contrast in the copper elemental mapping was limited, whereas the oxygen elemental mapping clearly revealed a significantly elevated concentration within oxidized regions, confirming successful selective oxidation.

In order to investigate the morphological differences between copper oxide and copper, AFM was employed to characterize them. Figure 1e presents the three-dimensional AFM image of the patterned substrate. A light blue dashed line separates the unoxidized region (left) from the oxidized region (right). The unoxidized region on the surface exhibited a smooth surface with minimal fluctuation. In contrast, the oxidized region on the right side displayed a burr-like structure, resulting in a significantly rough surface. The average Ra value of the oxidized region was 0.053 μm, compared to 0.012 μm for the unoxidized regions. This difference in surface roughness led to notable changes in the graphene surface morphology after hydrogen annealing. Figure 1f illustrates the surface morphology of the substrate after annealing, where the average Ra of the copper oxide region decreased to 0.041 μm. In high-temperature hydrogen environments, copper oxide undergoes the following reduction reactions:

H_2_ + 2CuO → H_2_O + Cu_2_O, (1)

H_2_ + Cu_2_O → H_2_O + 2Cu, (2)

H_2_ + CuO → H_2_O + Cu. (3)

Throughout H_2_ reduction, surface Cu atoms are transformed through Cu^2+^ → Cu^1+^ → Cu^0^ transitions [24]. The reduction reaction progressively increased the oxygen vacancy concentration as hydrogen continuously removed oxygen atoms. Upon reaching a critical vacancy threshold, the copper oxide surface structure collapsed, forming hole structures [25]. We used larger sample areas to facilitate morphological characterization. The three-dimensional holes (indicated by red arrows) were unfavorable for graphene nucleation due to the increased nucleation barrier [23,26]. Meanwhile, the unoxidized region maintained low roughness, with an average Ra of 0.015 μm after annealing. The self-limited growth behavior of graphene was feasible in the unoxidized region.

### 3.2. Regulation of Oxidation Time and Boundary Precision Between Oxidized and Unoxidized Regions

The above results indicate the critical role of selective substrate oxidation in the growth of patterned graphene. The accuracy of the oxidation process can significantly affect the precision of the patterned graphene.

**Figure 2:**
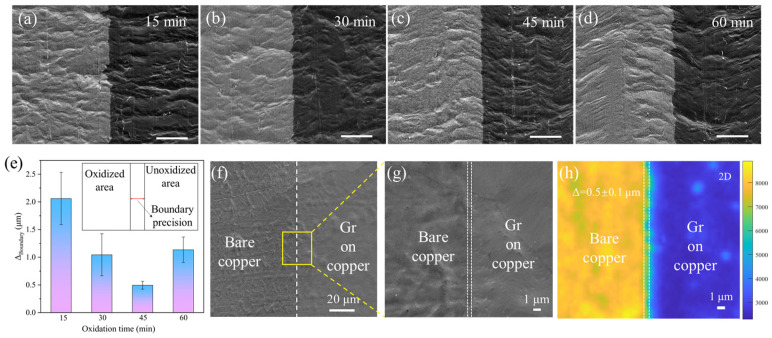
(**a**–**d**) SEM images of selectively oxidized copper substrates after oxidation for 15, 30, 45, and 60 min, respectively, scale bar: 10 μm; (**e**) histogram of variation in boundary precision via oxidation time; (**f**) SEM image of patterned graphene grown on copper oxidized for 45 min; (**g**) magnified SEM image of boundary in (**f**); (**h**) Raman 2D peak mapping of graphene corresponding to (**g**).

In this study, we employed low-concentration hydrogen peroxide (5% in DI water) for precise control under varying durations. Figure 2a–d show SEM images of copper substrates subjected to different oxidation durations (detailed in Materials and Methods). For oxidation times below 45 min, the precision of the boundary increased with time. At 15 min, the boundary exhibited precision of 2 ± 0.5 μm. When extended to 30 min, the precision increased to 1 ± 0.4 μm. At 45 min, the boundary precision reached an optimal value of 0.5 ± 0.1 μm. However, further extension to 60 min resulted in diminished precision, decreasing to 1.1 ± 0.2 μm. A histogram of the variation in boundary precision is presented in Figure 2e, with the corresponding statistical data presented in Table 1.

At the photoresist–copper interface, aqueous hydrogen peroxide oxidized copper regions not covered by the photoresist. During shorter oxidation periods (<45 min), incomplete oxidation at the photoresist–copper boundary resulted in irregular edges, as illustrated in Figure 2a,b. When the oxidation time reached 45 min, the hydrogen peroxide achieved complete oxidation precisely along the photoresist–copper interface, forming exceptionally sharp and well-defined edges. However, extending oxidation to 60 min allowed hydrogen peroxide to diffuse beneath the photoresist, creating irregular oxidation fronts that consequently degraded the edge precision. An oxidation time of 45 min was found to be optimal in our work.

Based on a high-precision patterned substrate, the high-precision patterned graphene was then synthesized in situ. Figure 2f presents an SEM image of patterned graphene on copper baked in air 120 °C for 10 min (air oxidation is specifically used here to differentiate from previous descriptions). Without graphene growth, air oxidation induced the formation of copper oxide structures, resulting in a rough surface, whereas the graphene-covered regions remained highly smooth. A higher-magnification SEM image demonstrating this is shown in Figure 2g. The corresponding Raman 2D peak mapping (Figure 2h) exhibited boundary precision of 0.5 ± 0.1 μm, consistent with previous SEM statistics.

### 3.3. Growth of Patterned Graphene Based on Optimized Patterned Substrate

The above results confirmed the presence of a patterned substrate with the optimized oxidation time of 45 min. Based on this, we systematically investigated the growth process of graphene in the selectively oxidized copper substrate.

**Figure 3:**
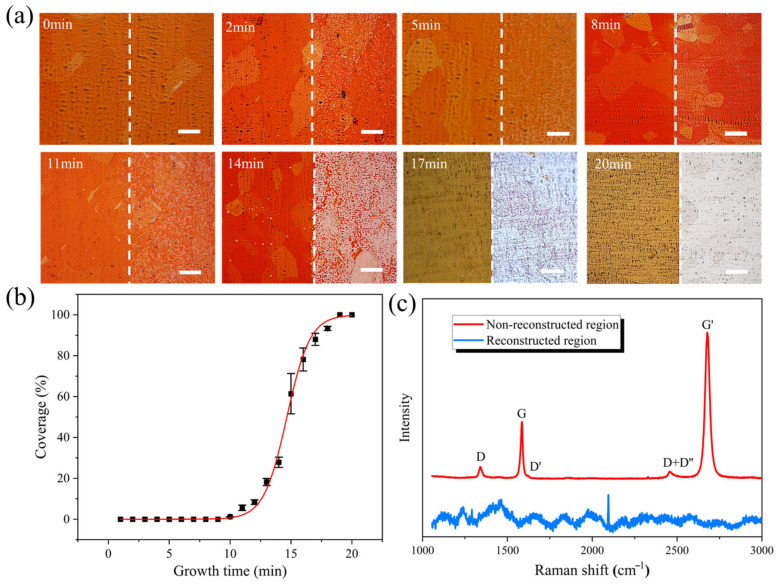
(**a**) Optical micrographs of patterned graphene growth with different growth times, scale bar: 50 μm; (**b**) fitting curve of graphene coverage varying with growth time in unoxidized region; (**c**) Raman spectra of patterned graphene transferred to SiO_2_ (D peak of ~1342 cm^−1^, G peak of ~1580 cm^−1^, D+D’’ peak of ~2460 cm^−1^, G’ peak of ~2700 cm^−1^).

Figure 3a presents optical microscopy images of graphene grown on the copper substrate at different growth durations. The oxidized (left) and unoxidized (right) regions are demarcated by white dashed lines. Prior to imaging, all samples were assessed in air oxidation to visualize the patterned graphene. Throughout the growth period, no visible graphene domains were observed in the oxidized region. In contrast, the unoxidized region exhibited a low nucleation density and limited graphene coverage during the early stages of growth. The domain size increased significantly to 10 μm by 14 min. Extending the growth time to 17 min achieved ≈70% coverage, ultimately reaching 100% monolayer coverage at 20 min, in agreement with reported graphene growth kinetics [27]. Figure 3b quantifies the evolution of graphene coverage in the unoxidized region through statistical analysis. The fitted curve follows the Boltzmann growth model, consistent with the self-limiting growth mechanism of graphene [28,29].

To evaluate the quality of the obtained patterned graphene, samples grown for 20 min were transferred to a SiO_2_/Si substrate for Raman spectroscopy analysis. Figure 3c presents the Raman spectra of the patterned graphene. In the unoxidized region, the G peak at approximately 1580 cm^−1^ and the G’ peak at approximately 2700 cm^−1^ exhibited a single Lorentzian peak shape, indicating the good crystallinity of the graphene [30,31]. Furthermore, the intensity ratio of the G’ peak to the G peak was approximately 2:1, suggesting the strong monolayer nature of the material [32,33,34,35]. The D and D’ peaks were defect-related Raman modes in graphene, which arose from the polycrystalline nature of the graphene. The D+D’’ peak originated from intervalley phonon scattering in graphene [35]. In contrast, no characteristic graphene peaks were observed in the oxidized regions, confirming the absence of graphene growth.

### 3.4. Fabrication of Flexible Electronics Based on Patterned Graphene

**Figure 4:**
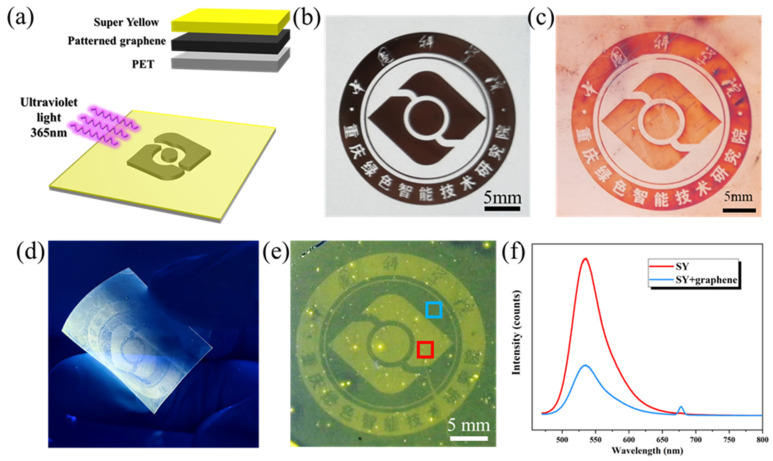
(**a**) Schematic diagram of graphene-based flexible electroluminescent device structure; (**b**) mask plate of Chongqing Institute logo and (**c**) corresponding patterned graphene on copper; (**d**) pattern graphene transferred onto flexible PET; (**e**) flexible graphene photoluminescent device under ultraviolet illumination, with the blue box and the red box correspond to the graphene-covered area and the uncovered area, respectively; (**f**) photoluminescence spectrum of corresponding regions in (**e**). In the institute logo in (**b**,**c**,**e**), the upper part of the outer ring displayed the Chinese characters for “Zhong Guo Ke Xue Yuan”, while the lower part displayed “Chong Qing Lv Se Zhi Neng Ji Shu Yan Jiu Yuan”, with the central point of the circle serving as the dividing boundary.

Super Yellow (SY), a widely used emissive layer in OLEDs due to its strong fluorescence [36,37], was employed to demonstrate the photoluminescence properties of flexible graphene electronics. As illustrated in Figure 4a, SY dissolved in toluene was spin-coated (800 rpm, 45 s) onto graphene-transferred PET substrates. The inset in Figure 4a depicts a cross-sectional schematic diagram of the device architecture, with the layered structure comprising SY, graphene, and the PET substrate sequentially arranged from top to bottom. The Chongqing Institute logo photolithography mask (Figure 4b) generated corresponding patterned graphene on copper (Figure 4c).

Figure 4d shows the fabricated flexible graphene-based luminescent device under 365 nm UV irradiation. Graphene can strongly quench the emission of nearby dye molecules through energy transfer, which is favorable for the patterned flexible luminescent device. The fluorescence quenching mechanism of graphene arose from the enhanced non-radiative decay of excited fluorophores. Under the energy transferring between fluorophores and quenchers, fluorophore emission was efficiently suppressed. Graphene serves as a unique interfacial platform for Förster resonance energy transfer (FRET) due to its electronic properties as a zero-gap semimetal with near-universal optical transparency [38,39]. Its linear band dispersion and near-constant optical absorption at Brillouin zone corners enable the quenching of photoexcited states through resonant energy transfer via electron–hole pair excitation [40,41]. The institute logo remained distinctly visible on the bent PET substrate: graphene-free regions displayed enhanced fluorescence, whereas fluorescence quenching occurred in graphene regions. The fluorescence spectra of the graphene-covered and graphene-free regions in Figure 4e were measured. In Figure 4f, the red and blue curves correspond to the regions in Figure 4e. The SY layer exhibited a fluorescence excitation peak at 535 nm (450 nm excitation), with the fluorescence intensity in graphene-free regions (*I*_Gr-free_) and graphene-covered regions (*I*_Gr-cov_) showing a ratio ≈3. The π-conjugated structure of the SY layer enabled π–π stacking interactions with graphene, leading to electron transfer-mediated fluorescence quenching [42,43]. The weak peak observed at 675 nm may have resulted from interference effects occurring when both the excitation light and emitted light interacted on the same side of the sample.

## 4. Conclusions

In summary, we successfully synthesized patterned graphene with high precision by substrate surface oxidation technology. By regulating the oxidation time, we precisely controlled the oxidation degree of the substrate and characterized the boundary precision between oxidized and non-oxidized regions. At an oxidation duration of 45 min, we achieved the high-precision in situ fabrication of patterned graphene on selectively oxidized substrates. Raman spectroscopy and 2D peak mapping of the graphene exhibited a low defect density with a spatial resolution of 0.5 ± 0.1 μm. The graphene growth process analysis indicated that the graphene coverage varied with the growth time in the unoxidized region, following the Boltzmann growth model, consistent with the self-limiting growth mechanism of graphene. Moreover, the flexible photoluminescent device composed of patterned graphene and SY demonstrated the fluorescence quenching effect of graphene, exhibiting a fluorescence intensity ratio of 3 between graphene-free regions and graphene-covered regions. These findings demonstrate that substrate surface oxidation affects patterned graphene’s growth and provides a contamination-free and damage-free fabrication route for high-precision graphene-based flexible electronics.

## Figures and Tables

**Table 1:** Summary of the boundary precision variation with different oxidation times.

Oxidation Time (min)	No. 1 * [μm]	No. 2 [μm]	No. 3 [μm]	No. 4 [μm]	No. 5 [μm]	Mean [μm]	Standard Deviation [μm]	Median [μm]
15	2.300	1.503	2.402	2.500	1.600	2.061	0.4717	2.30
30	0.889	1.000	1.482	1.334	0.519	1.045	0.3800	1.00
45	0.534	0.481	0.560	0.379	0.517	0.494	0.0705	0.52
60	1.446	1.154	1.250	0.962	0.865	1.135	0.2308	1.15

* Serial number refers to the order of measurements.

## Data Availability

The original contributions presented in this study are included in the article. Further inquiries can be directed to the corresponding author.
